# Potential prognostic and therapeutic value of ANXA8 in renal cell carcinoma: based on the comprehensive analysis of annexins family

**DOI:** 10.1186/s12885-023-11165-x

**Published:** 2023-07-18

**Authors:** Li-Hui Wang, Bo Cao, Yun-Long Li, Bao-Ping Qiao

**Affiliations:** 1 Department of Urology Surgery, The First Affiliated Hospital of Zhengzhou University, Zhengzhou, 450000 China; 2 Department of Emergency Surgery, Zhengzhou Central Hospital Affiliated to Zhengzhou University, Zhengzhou, 450000 China

**Keywords:** Annexins, Renal cell carcinoma, ANXA8, Cell cycle

## Abstract

**Background:**

Annexins are a family of proteins involved in a wide variety of cellular processes such as inflammation, proliferation, differentiation, apoptosis, migration and membrane repair. However, the role of most Annexins in renal cell carcinoma (RCC) remained unclear.

**Methods:**

The differentially expressed Annexins in RCC compared with normal controls were screened applying the TCGA database. The correlation of differentially expressed Annexins with clinical stages, grades and overall survival was analyzed to explore the clinical significance of Annexins in RCC. Then ANXA8 was selected and further stained in the discover and validation RCC cohort. The correlation of ANXA8 expression with clinical parameter was verified at the protein level. To explore the potential function of *ANXA8*, *ANXA8* was knockdown in the RCC cell line and further analyzed using transcriptome and bioinformatic analysis.

**Results:**

mRNA expression of *ANXA1*, *ANXA2R*, *ANXA4*, *ANXA8*, *ANXA8L1* and *ANXA13* were significantly upregulated in RCC compared with normal kidney tissues. In contrast, *ANXA3* and *ANXA9* mRNA expression was significantly downregulated. Higher expression of *ANXA2R*, *ANXA8* and *ANXA8L1* were correlated with worse overall survival, while lower expression of *ANXA3*, *ANXA9* and *ANXA13* were associated with worse clinical outcomes in RCC patients. We further demonstrated that ANXA8 expression was significantly increased in RCC compared with normal renal tissues at the protein level. And higher protein expression of ANXA8 was associated with higher clinical grades. Through the bioinformatics analysis and cell cycle analysis, we found knockdown of *ANXA8* mainly influenced the cell cycle and DNA replication. The top ten hub genes consist of *CDC6*, *CDK2*, *CHEK1*, *CCNB1*, *ORC1*, *CHEK2*, *MCM7*, *CDK1*, *PCNA* and *MCM3*.

**Conclusions:**

Multiple members of Annexins were abnormally expressed and associated with the prognosis of RCC. The expression of ANXA8 was significantly increased in RCC and associated with poor prognosis. ANXA8 might influence the cell cycle and could be a potential biomarker and therapeutic target for RCC.

**Supplementary Information:**

The online version contains supplementary material available at 10.1186/s12885-023-11165-x.

## Introduction

Renal cell carcinoma (RCC) refers to a heterogeneous group of urological neoplasm which is derived from renal-epithelial cell. RCC remains one of the most lethal urological malignant cancer worldwide, with up to 17% of patients developing metastases at the time of diagnosis [1] and a 5-year survival rate of 12% for late-stage RCC patients. Despite the improvement of management (for instance the immunotherapy strategies [2]) in RCC, there is still an urgent need to identify more potentially effective therapeutic biomarkers and targets for RCC.

Annexins are a family of proteins that share unique Ca^2+^ dependent phospholipids binding modules with conserved four or eight annexin repeats which enable them to bind with cellular membranes. Given its Ca^2+^ dependent roles interacting with cellular membranes, Annexins were involved in a wide variety of cellular processes such as inflammation, apoptosis, endocytosis, ion channel regulation, membrane organization and traffic [3]. The N-terminal domain of Annexins varied greatly between families conferring it with distinct functions. Accumulating studies revealed that Annexins were implicated in the development and progression of different types of cancers. Their expression was found differentially expressed in a broad spectrum of cancers covering reproductive, digestive, respiratory, urinary, nervous, hemopoietic and immune systems, etc. [4], making Annexins potential tumor biomarkers. Besides, Annexins also participated in the process of cell proliferation, metastasis and multidrug resistance of certain cancers, making Annexins promising therapeutic targets [5, 6].

Previous studies have found increased expression of *ANXA1*, *ANXA2* and *ANXA5* in RCC compared with non-neoplastic kidney. Upregulated expression of *ANXA1* [7], *ANXA2* [8–10] and *ANXA5* [11] could either affect cell migration, invasion or cell proliferation and was associated with poor prognosis of RCC. Meanwhile, studies also found that *ANXA3* [12] and *ANXA4* [13] were differentially expressed in RCC.

However, the roles and mechanisms of most Annexins remained unclear. *ANXA8* is a member of the Annexins family. Previous studies confirmed that *ANXA8* is upregulated in numerous types of malignant tumors including cholangiocarcinoma [14], breast cancer [15], pancreatic cancer [16], bladder cancer [17] and ovarian cancer [18]. However, its specific role and relevant mechanism in RCC have not been elucidated. To gain insight into the putative role of the Annexins family, especially *ANXA8* in RCC, we preliminarily screened the differentially expressed genes of the Annexins family between RCC and normal kidney tissues using TCGA database and analyzed their correlation with clinical prognosis. Further experiments were performed to verify the clinical significance and explore the mechanism of *ANXA8* in RCC.

## Methods

### TCGA analysis

The transcriptome profiling and corresponding clinical information for RCC (including renal clear cell carcinoma, renal papillary cell carcinoma, kidney chromophobe and kidney sarcoma) was retrieved from the open-source TCGA database (http://xena.ucsc.edu/welcome-to-ucsc-xena/). All the following data processing was implemented by R software. Original gene expression data was normalized using the trimmed mean of M-values method and the edgeR package was applied to figure out the differentially expressed genes (DEGs). Genes with the absolute value of Log_2_ fold change ≥ 1.0 and false discovery rate (FDR) value < 0.05 were regarded as statistically significant.

#### Clinical sample

Patients were divided into discover cohort and validation cohort. In the discover cohort, 30 patients with biopsy-proven RCC were enrolled from June 2018 to January 2019. Among the 30 RCC specimens, 21 of them had the corresponding normal portion of renal tissues obtained from the opposite pole of RCC. In the validation cohort, 40 patients with biopsy-proven RCC were enrolled from February 2023 to April 2023. All the samples were obtained from surgical operations performed in the Department of Urology, The First Affiliated Hospital of Zhengzhou University and examined by pathology experts to establish a definitive diagnosis. This study was approved by the Institutional Ethics Review Board of The First Affiliated Hospital of Zhengzhou University, and informed consent were obtained from all participants. All methods were carried out in accordance with relevant guidelines and regulations.

### Immunohistochemical staining of ANXA8

For immunohistochemical staining of ANXA8, specimens were fixed in the formalin for one week and embedded in the paraffin, cutting into 4 μm-thick sections. After de-paraffinization, hydration and subjecting to pressure cooker-based antigen retrieval in EDTA buffer, the sections were blocked by 3% bovine serum albumin and incubated with anti-ANXA8 antibody (1:100, ab111693, Abcam, Cambridge, UK) at 4 °C overnight. Then anti-rabbit secondary antibody (PV-9001, ZSBIO, Beijing, China) were used, followed by color development using 3, 3′-diaminobenzidine (ZLI-9018; ZSBIO). Finally, the images under 200 magnification were analyzed by Image-Pro Plus and presented as integrated optical density (IOD) to relatively quantify the expression of ANXA8.

### Cell culture

ACHN, CAKI-1, 769-P and 786-O were obtained from ATCC (Va, USA) and used within twenty times of passages. Human renal proximal tubular epithelial cells (HPTEC) were acquired from ScienCell (ScienCell, CA, USA) and made use of within passage five. The ACHN and CAKI-1 were cultured in Dulbecco’s modified Eagle medium (Gibco, MA, USA). 769-P and 786-O were cultured in RPMI 1640 medium (Gibco, MA, USA) and HPTEC were cultured in Epithelial Cell Medium (ScienCell, CA, USA). All the mediums were supplemented with 10% fetal bovine serum and 1% penicillin–streptomycin. Cells were seeded in a T25 flask or 6-well plate at 37 °C in a 5% CO_2_ humidified incubator and collected for further experiments when reaching approximately 80–85% confluence.

### Western blotting

Western blot was performed to compare the expression of ANXA8 in different cell lines and verify the knockdown effect of ANXA8. The cells were lysed in RIPA buffer supplemented with protease inhibitors (P2714, Merck, NJ, USA) to prepare for the extraction of cell protein and BCA Protein Assay Reagent Kit (23,225, Thermo Fisher, MA, USA) was applied to determine the protein concentration. Then 20 µg of protein loaded on each lane were separated by 12% SDS-PAGE gels and blotted onto PVDF membranes. The membranes were blocked in 5% non-fat dry milk in TBST at room temperature for 1 h and then incubated with primary antibody (anti-ANXA8 antibody (ab111693, Abcam, Cambridge, UK) and anti-β-actin antibody (ab8227, Abcam, Cambridge, UK) at 4 °C overnight. After that, the membranes were washed three times with TBST for 30 min and then incubated with HRP-conjugated secondary antibody at room temperature for 1 h. The immunoreactive bands were imaged and then analyzed by Photoshop. The densitometric values were normalized relative to the β-actin protein expression level.

### Quantitative real-time polymerase chain reaction (qRT-PCR)

Cells were harvested and total RNA was extracted using TRIzol reagent (Invitrogen, MA, USA) according to the manufacturer’s instruction. Then the purity and concentration of the RNA were quantified using Nanodrop (ND-1000, Thermo Fisher, MA, USA) and the same amount of RNA in each sample was reversely transcribed into cDNA with High Capacity cDNA Reverse Transcription Kits (Applied Biosystems, MA, USA). The qRT-PCR analysis was conducted using SYBR green Master Mix (Applied Biosystems) performed on ABI Prism 7500 sequence detection system (Applied Biosystems) with the following primer sequences: *ANXA8*-forward: GCTTAGGAACCAAGGAGGGT, *ANXA8*-reverse: AAGCTGCTCACATCATCCCT, *18 S*-forward: GTAACCCGTTGAACCCCATT, *18 S*-reverse: CCATCCAATCGGTA- GTAGCG, *β-actin*-forward: CATGTACGTTGCTATCCAGGC, *β-actin*-reverse: CTCCTTAATGTCACGCACGAT. Relative gene expression was normalized to *18 S* or *β-actin* rRNA. Data were analyzed using the comparative threshold cycle method and presented as relative fold change compared with the control group.

### Construction of stable ***shANXA8*** cell line

The plasmid construction, validation, lentivirus packaging and cell transfection were performed by Ji-Hua Biotechnology Services Co., Ltd (Beijing, China). In brief, a lentivirus vector targeting *ANXA8* was constructed and the lentivirus expressing scrambled shRNA was used as a negative control. Then lentivirus was packaged by transfecting 293T cells of 80-90% confluence. 293T cells were cultured for another 2–3 days and harvested by centrifugation at 1,000 × rpm for 5 min at 4 °C and filtered through a 0.45-µm filter. Subsequently, the supernatant containing lentiviral particles was centrifuged at 50,000 × g for 2 h at 4 °C and the lentivirus was collected. Next the 769-P cell was recovered and passaged 2–3 times to ensure cell viability. Each lentivirus vector was transfected into 769-P cells cultured in 6-well plates at a multiplicity of infection of 50. 48 h after transfection 8ug/ml puromycin was added for screening and the surviving cells were further amplified to gain the stable knockdown cells.

### RNA isolation and sequencing

The RNA isolation and RNA-seq analysis were conducted by Novogene Co., Ltd (Beijing, China). Total RNA was extracted from two groups of 769-P cells (transfected with *ANXA8* shRNA and control shRNA lentivirus vector respectively) with three duplicates for each group. A total amount of 1 µg RNA per sample was used as input material for the RNA sample preparations. Differential expression analysis was performed using the DESeq2 R package. The *p* values were adjusted using the Benjamini & Hochberg method. Genes with a padj < 0.05 and the absolute value of Log_2_ fold change ≥ 0.5 found by DESeq2 were assigned as differentially expressed followed by Kyoto Encyclopedia of Genes and Genomes (KEGG) [19] and Gene Ontology (GO) analysis. The use of the KEGG database has been approved by Kanehisa Laboratories. Protein-protein interaction network was constructed using the STRING database as previously described [20]. The top 10 hub genes were screened according to the Cytohubba degree in the Cytoscape.

#### Cell cycle analysis

The stable sh*ANXA8* cell line and control cells were seeded in 6-well dish and harvested by 0.25% trypsin when reaching 80–85% confluence. Digested cells were washed using pre-cooled phosphate-buffered saline and incubated with propidium (P4170, Sigma, MA, USA) plus RNase (EN0531, Thermo Fisher, MA, USA) and Triton X-100 (T8787, Sigma, MA, USA) for 30 min. Flow cytometry was conducted with BD LSRFortessa Cell Analyzer (BD Biosciences, NJ, USA).

### Statistical analysis

Normally distributed quantitative data are presented as mean ± SEM and non-normally distributed data are displayed as median with interquartile range. Variables were analyzed using a *t*-test for normally distributed data or Mann-Whitney test for non-normal distribution as appropriate. Multi-groups comparison was analyzed applying One-way ANOVA with Bonferroni correction or the Kruskal-Wallis test with Dunn’s multiple comparisons test. The overall survival difference was estimated with the Kaplan–Meier analysis and the *p* value was calculated using the Log-rank test. Gene expression less than the median value was classified into “low expression” group. Otherwise would be classified into the “high expression” group. All figures and statistical analysis were performed based on R language (version 3.4.2) and GraphPad Prism 7 software. *p* < 0.05 was considered statistically significant.

## Results

### Bioinformatic analysis revealed several differentially expressed Annexins in RCC

**Fig. 1:**
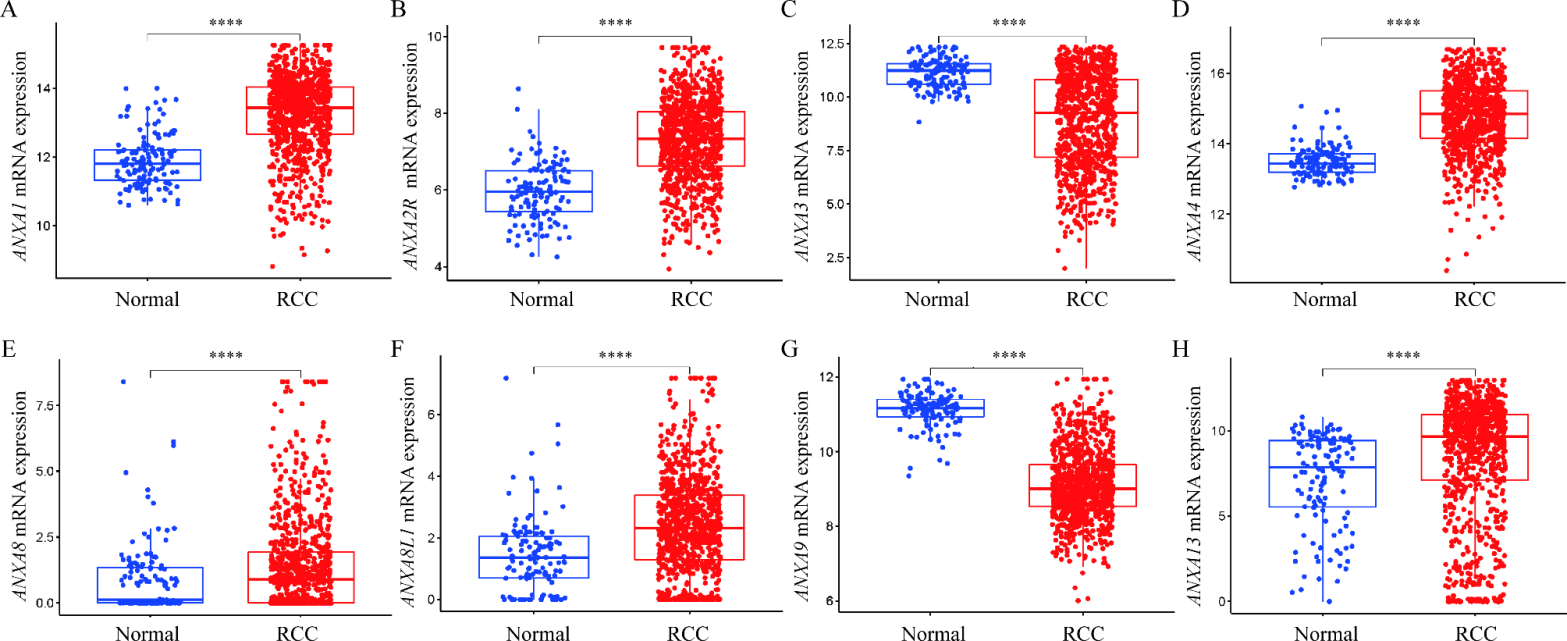
Bioinformatic analysis revealed several differentially expressed Annexins between normal and RCC tissues mRNA expression levels of **(A)***ANXA1*, **(B)***ANXA2R*, **(C)***ANXA3*, **(D)***ANXA4*, **(E)***ANXA8*, **(F)***ANXA8L1*, **(G)***ANXA9* and **(H)***ANXA13* in normal and RCC tissues. The mRNA expression levels were Log_2_ transformed. ****p < 0.0001 RCC, renal cell carcinoma

Based on the TCGA database we found 8 differentially expressed Annexins between RCC and normal kidney tissues, including *ANXA1*, *ANXA2R*, *ANXA3*, *ANXA4*, *ANXA8*, *ANXA8L1*, *ANXA9* and *ANXA13*. Among them, mRNA expression level of *ANXA1*, *ANXA2R*, *ANXA4*, *ANXA8*, *ANXA8L1* and *ANXA13* were significantly upregulated, while *ANXA3* and *ANXA9* were significantly downregulated in RCC compared with normal kidney tissues (Fig. 1).

### Relationship between differentially expressed Annexins with clinical parameters of RCC patients

**Fig. 2:**
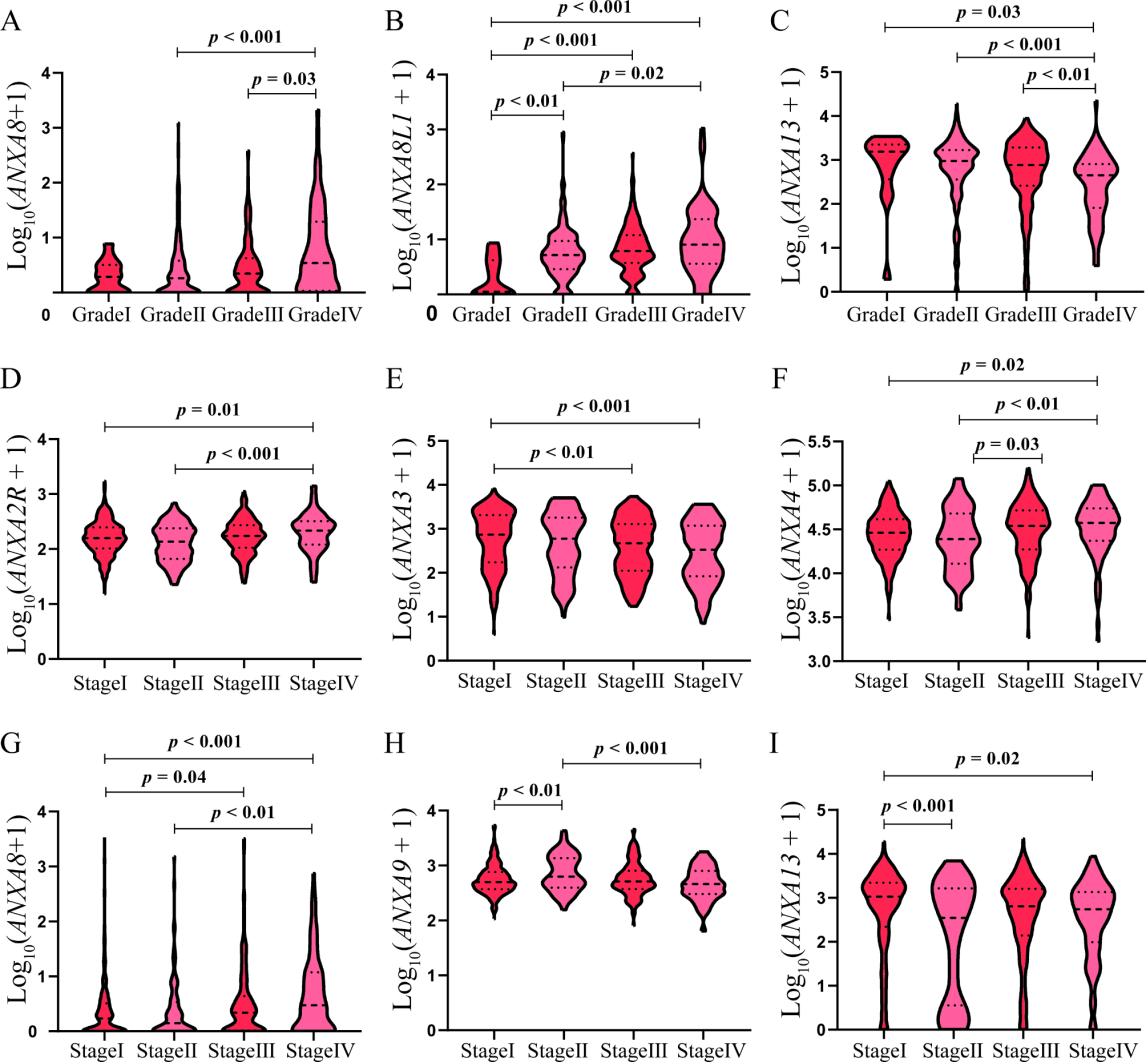
Relationship between differentially expressed Annexins with clinical parameters of RCC patients Comparison of expression levels of differentially expressed Annexins with clinical parameters in RCC (TCGA). **(A)***ANXA8*, **(B)***ANXA8L1* and **(C)***ANXA13* expression in different grades of RCC patients. **(D)***ANXA2R*, **(E)***ANXA3*, **(F)***ANXA4*, **(G)***ANXA8*, **(H)***ANXA9* and **(I)***ANXA13* expression in different stages of RCC patients. The mRNA expression levels were Log_10_ transformed RCC, renal cell carcinoma

To further explore the clinical significance of the 8 identified Annexins in RCC, we analyzed the correlation of the 8 DEGs expressions with RCC clinical grades and stages. Regarding the correlation with RCC grades, mRNA expression of *ANXA8* and *ANXA8L1* correlated positively with RCC grades (Fig. 2A-B), while *ANXA13* correlated negatively with RCC grades (Fig. 2C). Besides, mRNA expression levels of *ANXA2R*, *ANXA4 and ANXA8* were significantly higher in advanced RCC clinical stages (Fig. 2D F and 2G). However, mRNA expression levels of *ANXA3, ANXA9* and *ANXA13* were significantly higher in the early stages of RCC compared with those in advanced stages (Fig. 2E H and 2I).

### Differentially expressed Annexins were associated with the prognosis of patients with RCC

**Fig. 3:**
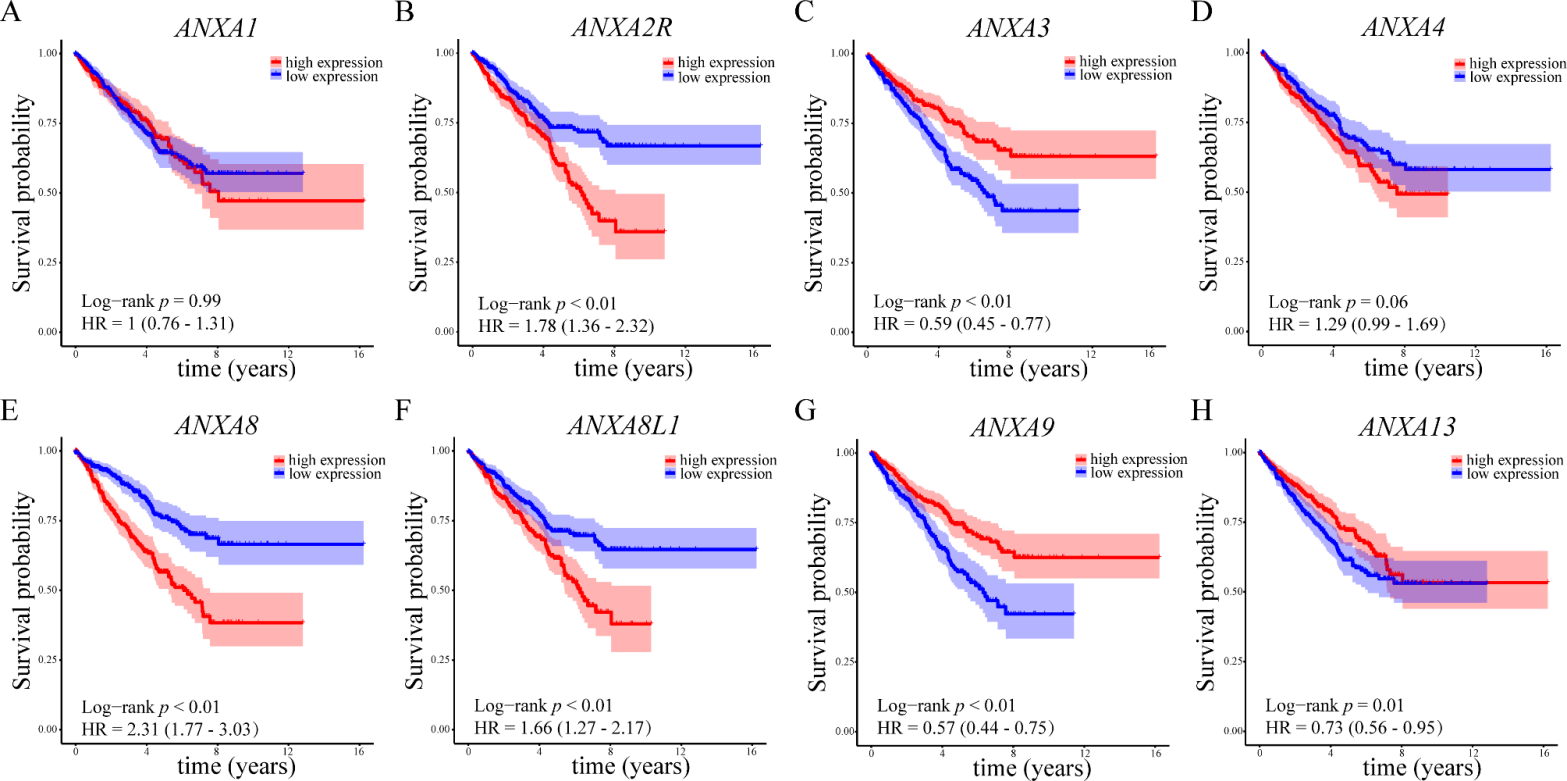
Differentially expressed Annexins were associated with prognosis of RCC patients Prognostic values of differentially expressed Annexins in RCC. Prognostic significance of **(A)***ANXA1*, **(B)***ANXA2R*, **(C)***ANXA3*, **(D)***ANXA4*, **(E)***ANXA8*, **(F)***ANXA8L1*, **(G)***ANXA9* and **(H)***ANXA13* in RCC. The *p*-value was calculated using the Log-rank test RCC, renal cell carcinoma; HR, hazard ratio

We then analyzed the correlations between the mRNA expression of the 8 DEGs and overall survival of RCC patients using the Kaplan–Meier survival analysis. The survival analysis demonstrated that upregulated mRNA expression levels of *ANXA2R*, *ANXA8* and *ANXA8L1* were associated with worse overall survival. Downregulated expression of *ANXA3* and *ANXA9* were associated with worse overall survival and upregulated expression of *ANXA13* was associated with better overall survival in RCC (Fig. 3). To validate the prognosis value of Annexins, we confirmed the relationship between mRNA expression of the 8 DEGs with overall survival of RCC patients using the online Kaplan–Meier plotter. Except *ANXA4*, the analysis using Kaplan–Meier plotter showed consistent results with our analysis using the TCGA database (Supplementary Fig. 1). Taken together, these results indicated that Annexins were of great significance for assessing the prognosis of RCC.

### Validation of upregulated expression of ANXA8 and its relevance in RCC

Based on above analysis, we found higher expression of *ANXA8* correlated with both stages, grades and overall survival of RCC patients. From the Kaplan–Meier survival analysis plot we could recognize that the overall survival between high and low *ANXA8* expression was most striking (HR = 2.31), indicating a close relationship between *ANXA8* and the clinical outcome of RCC. Therefore we mainly focused on the role of *ANXA8* in RCC in the following study.

**Fig. 4:**
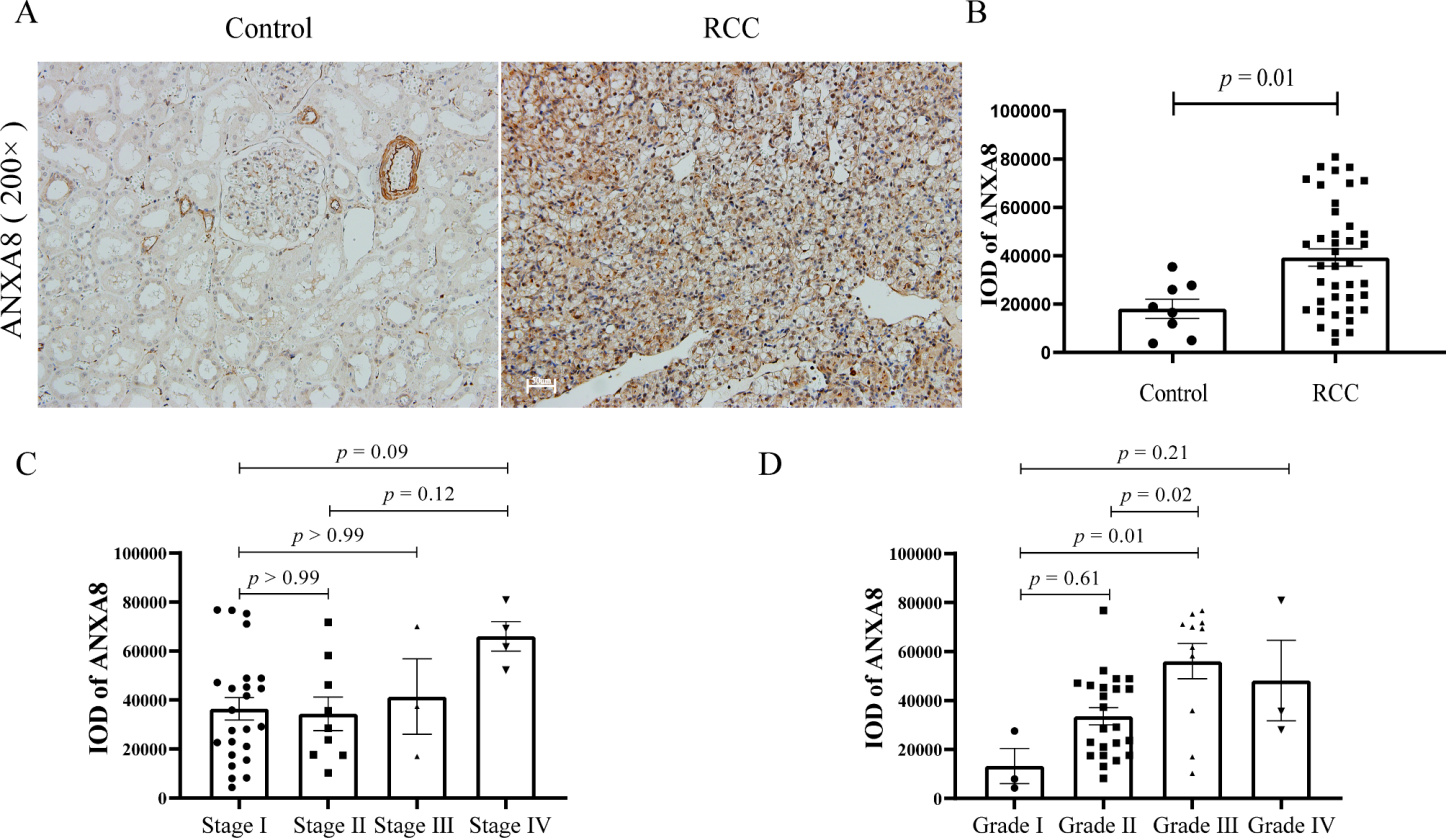
Validation of upregulated expression of ANXA8 and its relevance in RCC patients **(A)** Representative immunohistochemical staining of ANXA8 in normal and RCC tissues of validation cohort. Bar = 50 μm. **(B)** Semiquantitative analysis of the expression of ANXA8 in normal and RCC tissues of validation cohort. **(C)** ANXA8 expression of validation RCC patients in different clinical stages. **(D)** ANXA8 expression of validation RCC patients in different clinical grades IOD, integrated optical density; RCC, renal cell carcinoma

ANXA8 was immunohistostained in the discover cohort and validation cohort (the clinical parameters of which were shown in the Supplementary Tables 1 and Table 1 respectively). In normal renal tissues only blood vessels and few of the renal tubular epithelial cells were positively stained. While ANXA8 expression was significantly enhanced in RCC, mainly localized in the cytoplasm and cell membrane (Supplementary Fig. 2A-B, p < 0.001 and Fig. 4A-B, p = 0.01). Furtherly, we analyzed the correlation of ANXA8 expression and clinicopathological parameters in RCC patients. ANXA8 expression tend to be higher in the advanced stages and grades than stage I and grade I, while did not reach significance after multiple comparison correction (Supplementary Fig. 2C-D). When we enlarge the sample size in the validation cohort, ANXA8 expression was significantly elevated in grade III than grade I and II (Fig. 4C-D, p = 0.01 and *p* = 0.02 respectively), which verified the TCGA analysis. Besides, ANXA8 expression tend to be higher in stage IV compared to stage I and II while did not reach significance after correction.

**Table 1:** Clinical parameters of RCC patients in the validation cohort

Parameters	Cases (%)
Age (years)	
≤ 60	27 (67.5)
< 60	13 (32.5)
Gender	
Male	32 (80)
Female	8 (20)
Pathological subtype	
Clear cell renal carcinoma	40 (100)
TNM stage	
I	24 (60)
II	9 (22.5)
III	3 (7.5)
IV	4 (10)
Histological grade	
I	3 (7.5)
II	23 (57.5)
III	11 (27.5)
IV	3 (7.5)

### 769-P could better simulate the role of ***ANXA8*** in RCC

**Fig. 5:**
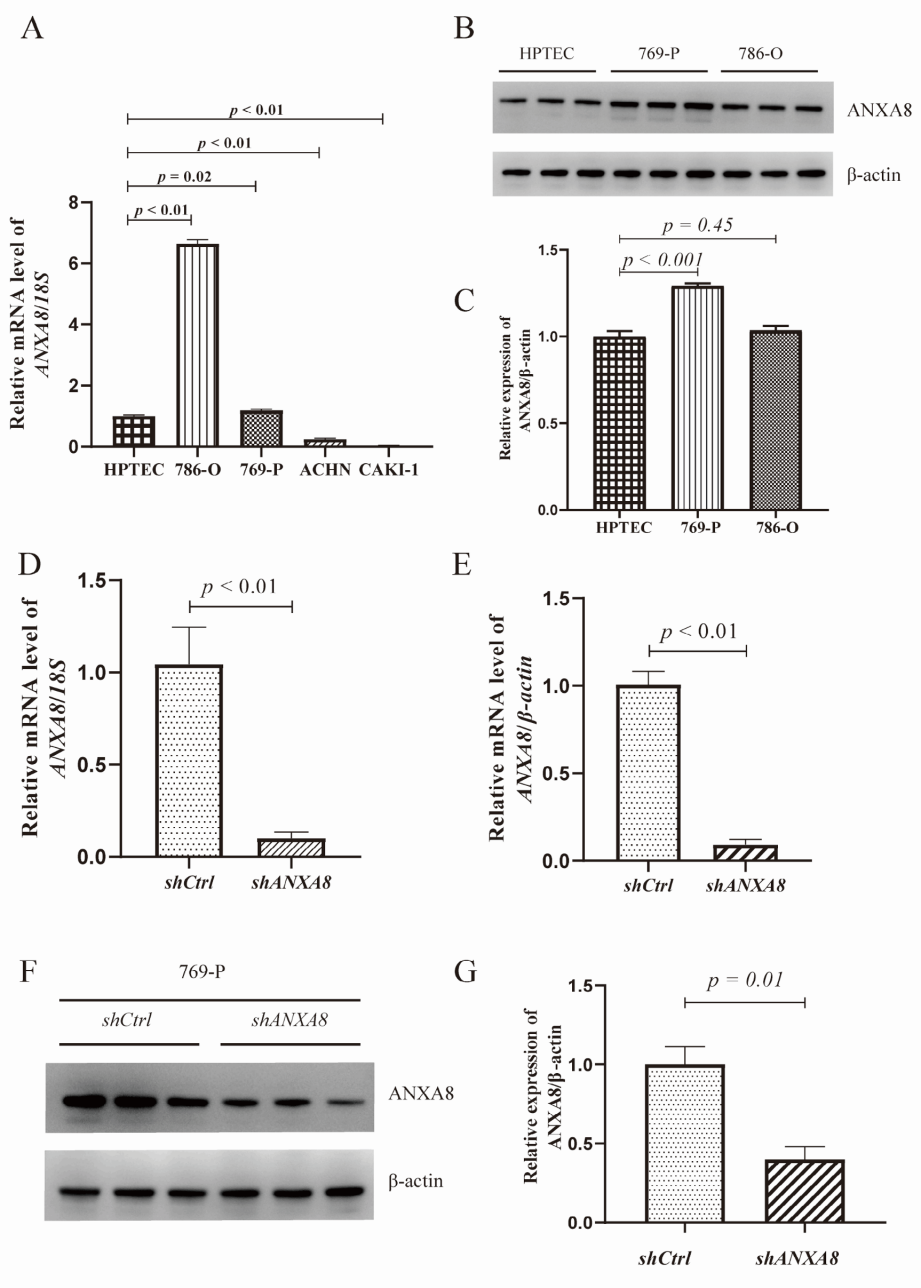
ANXA8 expression was increased in 769-P cell line **(A)** mRNA expression of *ANXA8* in HPTEC and four human RCC cell lines (786-O, 769-P, ACHN and CAKI-1). **(B)** Protein expression of ANXA8 in HPTEC, 769-P and 786-O. **(C)** Semiquantitative analysis of the relative protein expression of ANXA8 in HPTEC, 769-P and 786-O. The mRNA expression of *ANXA8* adjusted by **(D)***18 S* and **(E)***β-actin* in 769-P transfected with *shCtrl* and *shANXA8*. **(F)** Protein expression of ANXA8 in 769-P transfected with *shCtrl* and *shANXA8*. **(G)** Semiquantitative analysis of the relative protein expression of ANXA8 in 769-P transfected with *shCtrl* and *shANXA8* HPTEC, human renal proximal tubular epithelial cells; RCC, renal cell carcinoma

To explore the expression of *ANXA8* in RCC cell lines, we started with qRT-PCR performed in HPTEC and four human RCC cell lines (786-O, 769-P, ACHN and CAKI-1). As shown in Fig. 5A, mRNA expression of *ANXA8* was significantly upregulated in 786-O and 769-P, while downregulated in ACHN and CAKI-1 compared with HPTEC, indicating that 769-P and 786-O could better simulate the role of *ANXA8* in RCC patients at mRNA level. Next we investigated the protein expression of ANXA8 in HPTEC, 769-P and 786-O. However, the protein level of ANXA8 in 786-O displayed no difference compared with HPTEC. Only the 769-P RCC cell line displayed consistently elevated expression of ANXA8 at mRNA and protein levels compared with HPTEC (Fig. 5B-C). To assess the role of *ANXA8* in RCC, *ANXA8* was knockdown by transfection with lentivirus targeting *ANXA8* in 769-P (Fig. 5D-G).

### Knockdown of ***ANXA8*** mainly influenced the cell cycle

**Fig. 6:**
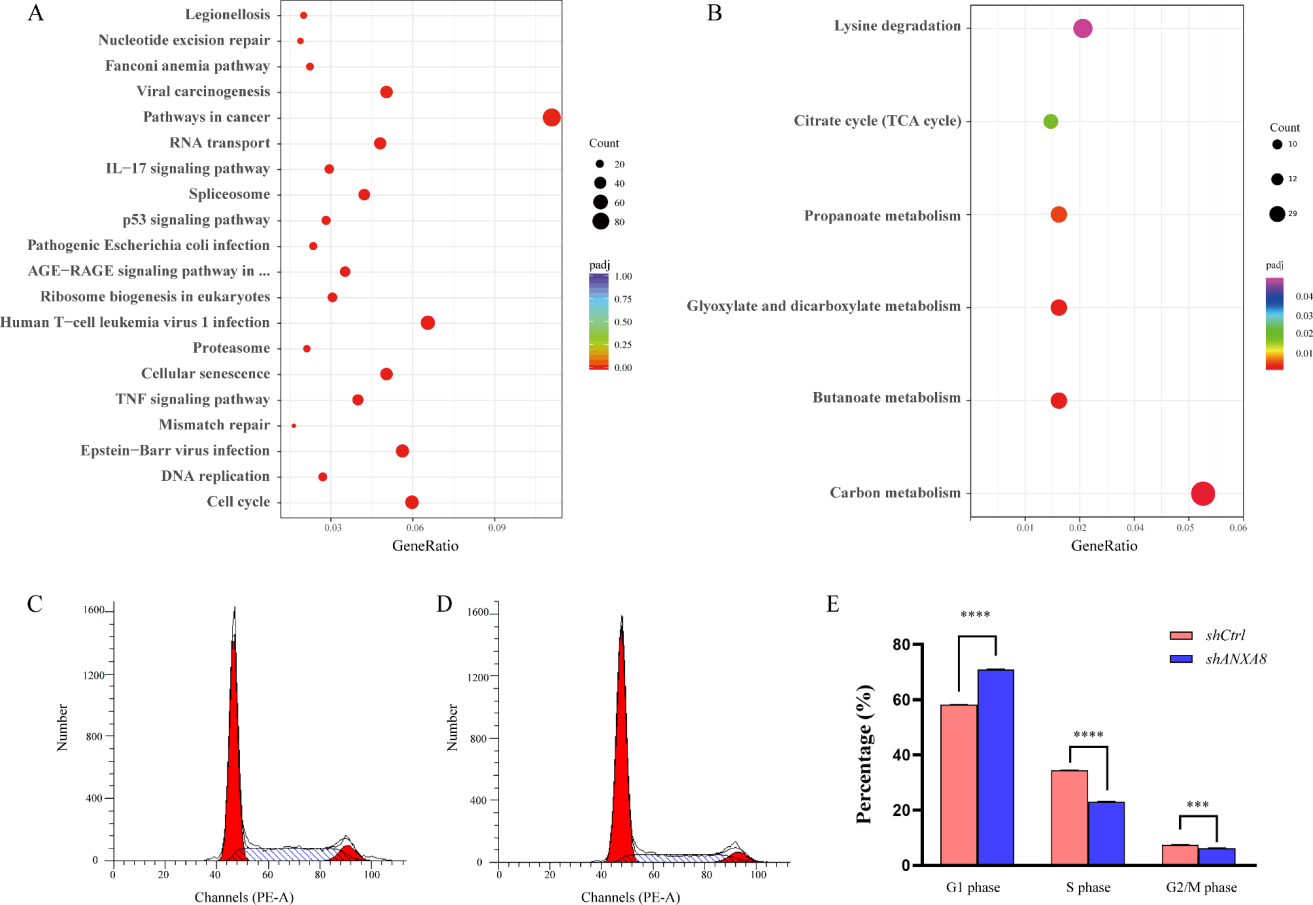
Knockdown of *ANXA8* mainly influenced the cell cycle in 769-P **(A)** Down and **(B)** up regulated KEGG pathways of the differentially expressed genes in 769-P transfected with *shANXA8* compared with *shCtrl*. **(C)** Representative cell cycle distribution by propidium staining in 769-P transfected with *shCtrl*. **(D)** Representative cell cycle distribution by propidium staining in 769-P transfected with *shANXA8*. **(E)** Quantitative analysis of proportion of the cells in different phases between 769-P transfected with *shANXA8* and *shCtrl*. ****p < 0.0001, ***p < 0.001

RNA sequencing was applied to explore the potential function of *ANXA8* in 769-P. 3577 genes were significantly differentially expressed in 769-P transfected with *shANXA8* compared with *shCtrl*. Among them, 1955 genes were downregulated and 1622 genes were upregulated (Supplementary Tables 2–3). DEGs were further analyzed with KEGG pathway analysis. The predominantly enriched pathways for the downregulated and upregulated DEGs were displayed in Fig. 6A-B. We found that the top 5 enriched pathways for downregulated DEGs were cell cycle, DNA replication, Epstein-Barr virus infection, mismatch repair and TNF signaling pathway. While the top 5 enriched pathways for upregulated DEGs mainly involved carbon metabolism, butanoate metabolism, glyoxylate and dicarboxylate metabolism, propanoate metabolism and citrate cycle (TCA cycle).

The GO enrichment analysis was further conducted through three aspects including biological process (BP), cellular component (CC) and molecular function (MF). The top 5 enriched BP, CC and MF terms for downregulated and upregulated DEGs were illustrated in Supplementary Fig. 3. It is interesting to note that DNA replication, DNA-dependent DNA replication, sister chromatid segregation, chromosome segregation and nuclear chromosome segregation for BP were significantly downregulated in *shANXA8* 769-P cells, suggesting knockdown *ANXA8* might influence the DNA replication.

To verify the effect of *ANXA8* on cell cycle and DNA replication, cell cycle was further analyzed by propidium staining in 769-P transfected with *shANXA8* and *shCtrl* (Fig. 6C-D). Quantitative analysis indicated that knockdown *ANXA8* reduced proportion of S phase and G2/M phase but increased G1 phase (Fig. 6E), indicating that knockdown *ANXA8* decreased DNA replication and mitosis.

### Identification of the hub genes related to ANXA8 in RCC

In order to screen out hub genes, protein-protein interaction network analysis was applied among the genes involved in the top 5 KEGG pathways. The top ten hub genes consisted of *CDC6*, *CDK2*, *CHEK1*, *CCNB1*, *ORC1*, *CHEK2*, *MCM7*, *CDK1*, *PCNA* and *MCM3* which was listed in Table 2.

**Table 2:** Hub genes identified by protein-protein interaction network analysis

node_name	Degree	Closeness	Radiality	Betweenness	Stress	Coefficient
*CDC6*	73	96.67	2.42	666.95	5272	0.53
*CDK2*	70	98.67	2.56	516.58	10,160	0.56
*CHEK1*	67	96.17	2.49	309.23	5352	0.60
*CCNB1*	64	95.67	2.52	306.28	5792	0.60
*ORC1*	64	92	2.34	459.17	3912	0.58
*CHEK2*	63	94.83	2.49	281.80	5562	0.63
*MCM7*	62	91	2.33	79.61	1958	0.70
*CDK1*	62	93.33	2.44	152.99	3300	0.65
*PCNA*	61	91	2.34	90.95	1990	0.70
*MCM3*	61	92.50	2.41	127.02	3000	0.69

## Discussion

To gain insight into the putative role of Annexins in RCC, we compared the mRNA expression of the genes related to Annexins family between RCC and normal kidney tissues based on the TCGA database, and further analyzed the correlation of the differentially expressed genes with clinical stages, histological grades and overall survival rate of RCC patients. One previous study by Wei et al. integrating the ONCOMINE database with other online bioinformatic analyses detected that a majority of Annexins members were dysregulated and could be a potential biomarker for progression and prognosis in clear renal cell carcinoma [21]. While our investigation using the TCGA database enhanced the credibility and provided a second filter for the significance of Annexins in RCC. Altogether we found 8 DEGs of Annexins were differentially expressed in RCC tissues compared with normal tissues, including *ANXA1*, *ANXA2R*, *ANXA3*, *ANXA4*, *ANXA8*, *ANXA8L1*, *ANXA9* and *ANXA13*.

Our analysis confirmed the research by Yamanoi et al. who found upregulated expression of ANXA1 in RCC [7]. Nevertheless, *ANXA1* expression was not associated with the overall survival of RCC patients. This could attribute to the sample size difference and contradictory role of *ANXA1* in regulating proliferation and tumor growth in cancer [22]. In addition to *ANXA1*, the remaining 7 DEGs could be divided into two groups.

The members of the first group are *ANXA2R*, *ANXA4*, *ANXA8* and *ANXA8L1*, which were upregulated in RCC and higher expression correlated with worse clinical outcomes, indicating a pathogenic role in RCC. So far *ANXA2* is the most studied Annexins in RCC^[9, 10]^. The reason why *ANXA2* was not differentially expressed in our analysis may lie in the screening standard of DEGs. Genes with |Log_2_ fold change | ≥ 1.0 and FDR value < 0.05 were regarded as statistically significant. Although *ANXA2* didn’t reach the above criteria, the expression of *ANXA2* increased by 1.8 times with FDR value < 0.05 in the RCC TCGA database. Additionally, increased expression of *ANXA2R*, which is a receptor of *ANXA2*, further supported the potential role of *ANXA2* in RCC. Besides, our results verified previous study by Zimmermann et al. who found increased protein expression of ANXA4 in RCC [13]. ANXA5 expression was previously found elevated at both mRNA and protein levels in 22 pairs of RCC tissues and high ANXA5 expression promotes tumor progression and poor prognosis in RCC [11]. However, our analysis using TCGA data could not support the results at mRNA level.

The second group consists of *ANXA3*, *ANXA9* and *ANXA13*. Downregulated expression of *ANXA3* and *ANXA9* were associated with worse clinical outcomes and upregulated expression of *ANXA13* was associated with better clinical outcomes in RCC, suggesting a protective role of *ANXA3*, *ANXA9* and *ANXA13* in RCC. A previous study by Bombelli et al. found *ANXA*3 could restrict lipid storage responsible for the adipocyte-like phenotype of clear cell RCC cells which might explain the protective mechanism [23]. Nevertheless in the few studies performed related to the effect of *ANXA9* and *ANXA13* in tumour demonstrated *ANXA9* and *ANXA13* could promote invasion and metastasis in a certain types of cancers [24–27]. Hence the function and mechanism of *ANXA9* and *ANXA13* in RCC might need further investigation.

Among the 8 DEGs, *ANXA8* was of special interest. According to the analysis we found higher expression of *ANXA8* correlated with both worse grades, stage and overall survival. From the Kaplan–Meier survival analysis plot we could recognize that the overall survival between high and low *ANXA8* expression was most striking (HR = 2.31), indicating a close relationship between *ANXA8* and RCC. Besides, higher expression of *ANXA8L1*, which is a paralog of *ANXA8*, also correlated with worse clinical outcomes of RCC. Therefore we mainly focused on *ANXA8* in the following experiment.

To validate the role of *ANXA8* in RCC, ANXA8 was immunohistostained in the discover and validation RCC patients and correlation of ANXA8 expression with clinicopathological parameters of RCC patients was analyzed. ANXA8 expression was significantly enhanced in RCC compared with normal renal tissues. Further correlation analysis manifested that higher expression of ANXA8 was associated with higher grades which verified the analysis in TCGA.

To explore the mechanism of *ANXA8* in RCC, *ANXA8* was knockdown by transfection with lentivirus targeting *ANXA8* in 769-P and RNA-sequencing was applied. A total of 3577 genes were significantly differentially expressed. Among them, 1955 genes were downregulated and 1622 genes were upregulated in 769-P transfected with *shANXA8* compared with *shCtrl*. DEGs were further analyzed with KEGG pathway analysis and GO enrichment analysis, both of which manifested that knockdown *ANXA8* mainly influenced cell cycle and DNA replication. This result was further corroborated by cell cycle analysis which demonstrated that knockdown *ANXA8* in 769-P promote S/G2 to G1 transition. Cell proliferation is tightly controlled under cycle phase transitions driven by the cell cycle, while cancer cells are characterized by unrestricted proliferation. The cell cycle is a finely regulated process mainly consisting of proteins called cyclins and their catalytic partners, the cyclin-dependent kinases (CDKs) [28]. Different cyclin-CDK complexes become orderly activated in specific phases of the cell cycle enabling proper replication of genomic DNA and cell division. Dysfunction of the cell cycle could lead to uncontrolled cell proliferation causing carcinogenesis [29, 30].

In order to screen out hub genes, protein-protein interaction network analysis was applied among the genes involved in the top 5 KEGG pathways. The top ten hub genes consisted of *CDC6*, *CDK2*, *CHEK1*, *CCNB1*, *ORC1*, *CHEK2*, *MCM7*, *CDK1*, *PCNA* and *MCM3*, among which *CDC6* has the highest node degree. *CDC6* is indispensable for the initiation of DNA replication. During G1 phase, CDC6 is recruited to the origin recognition complex (ORC) at the origins of DNA replication. Together with CDT1, the ORC-CDC6-CDT1 complex enable loading of the MCM proteins onto chromatin, licensing DNA replication [31]. *CDC6* is overexpressed in various malignant tumors including ovarian cancer [32], pancreatic cancer [33], osteosarcoma [34] and breast cancer [35], exerting an oncogenic effect. Interestingly, a recently published study demonstrated increased expression of CDC6 being a potential prognostic factor of poor prognosis in RCC patients [36]. However, considering the potential off-target effect of sh*ANXA8* in our study, more investigation is needed to confirm the mechanism of *ANXA8* in RCC.

In conclusion, multiple members of Annexins were abnormally expressed and associated with the prognosis of RCC. The expression of *ANXA8* was significantly increased in RCC and associated with poor prognosis. *ANXA8* might influence the cell cycle and could be a potential biomarker and therapeutic target for RCC.

## Electronic supplementary material

Below is the link to the electronic supplementary material.

Supplementary Material 1

Supplementary Material 2

Supplementary Material 3

Supplementary Material 4

## Data Availability

The raw transcriptome data in this work was submitted to NCBI with Bioproject ID PRJNA856171 which could be accessed at https://www.ncbi.nlm.nih.gov/bioproject/.

## Abbreviations

BP: Biological process
CC: Cellular component
CDKs: Cyclin-dependent kinases
DEGs: Differentially expressed genes
FDR: False discovery rate
GO: Gene Ontology
HPTEC: Human renal proximal tubular epithelial cells
IOD: Integrated optical density
KEGG: Kyoto Encyclopedia of Genes and Genomes
MF: Molecular function
ORC: Origin recognition complex
RCC: Renal cell carcinoma
