# Supplementary material for: Potential prognostic and therapeutic value of ANXA8 in renal cell carcinoma: based on the comprehensive analysis of annexins family
Source: BMC Cancer. 2023 Jul 18;23:674. doi: 10.1186/s12885-023-11165-x (PMC10355003; doi:10.1186/s12885-023-11165-x)
Supplement: Supplementary file 1 — Supplementary Material 1 [file 12885_2023_11165_MOESM1_ESM.docx]

**Supplementary Figure 1. Validation of the association of differentially expressed Annexins with prognosis of RCC patients using the online Kaplan–Meier plotter.**


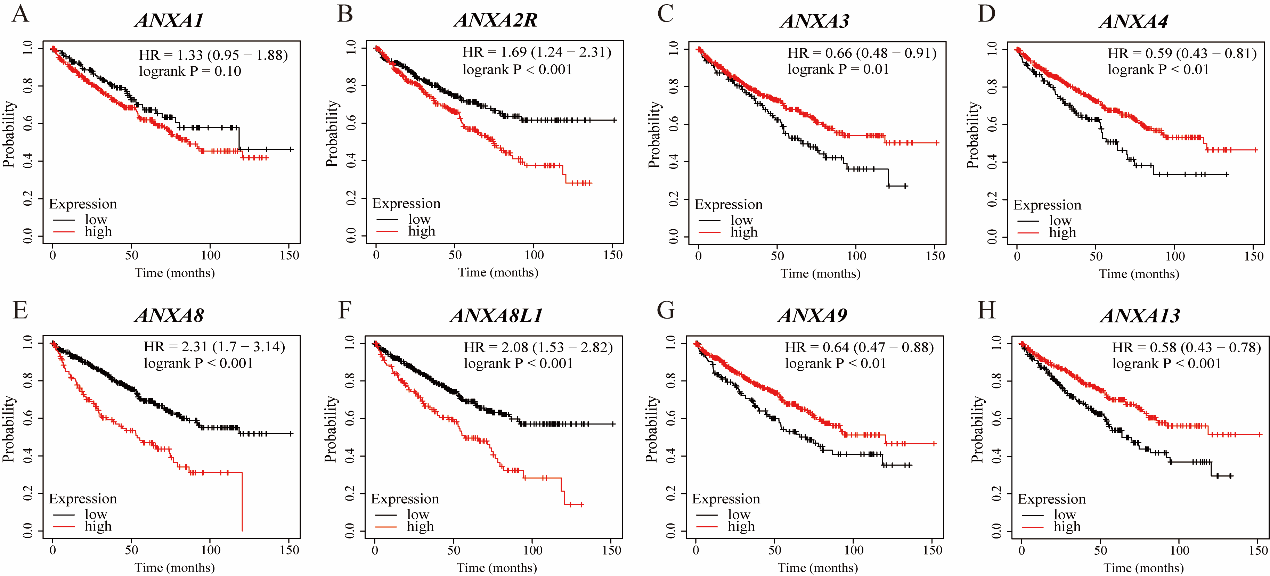


Prognostic significance of (A) *ANXA1*, (B) *ANXA2R*, (C) *ANXA3*, (D) *ANXA4*, (E) *ANXA8*, (F) *ANXA8L1*, (G) *ANXA9* and (H) *ANXA13* in RCC.

RCC, renal cell carcinoma; HR, hazard ratio

**Supplementary Figure 2. Validation of upregulated expression of ANXA8 and its relevance in the discover RCC patients.**

**
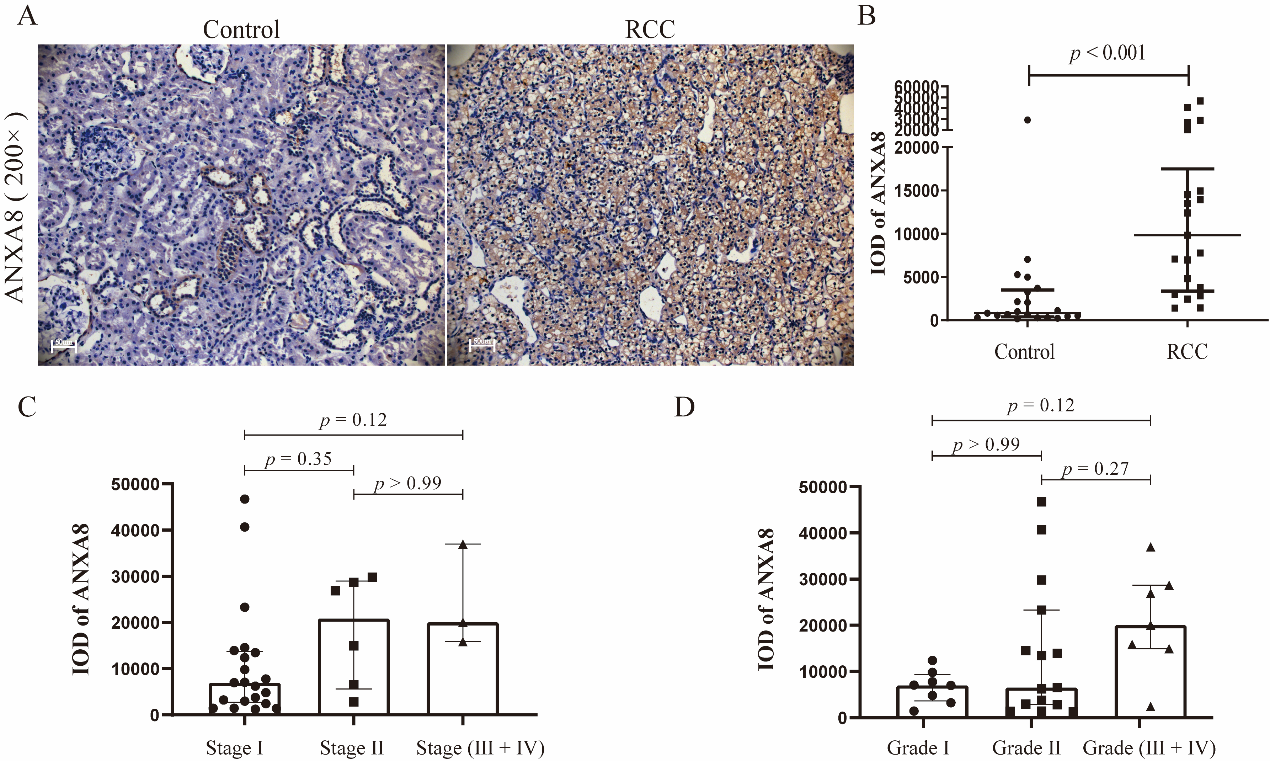
**

(A) Representative immunohistochemical staining of ANXA8 in normal and RCC tissues. Bar = 50 µm. (B) Semiquantitative analysis of the expression of ANXA8 in normal and RCC tissues in the discover cohort. (C) ANXA8 expression of the discover RCC patients in different clinical stages. Dunn’s test was applied for multiple comparison correction. (D) ANXA8 expression of the discover RCC patients in different clinical grades. Dunn’s test was applied for multiple comparison correction.

IOD，integrated optical density; RCC, renal cell carcinoma.

**Supplementary Figure 3. GO analysis of the differentially expressed genes in 769-P transfected with *shANXA8* compared with *shCtrl*.**

**
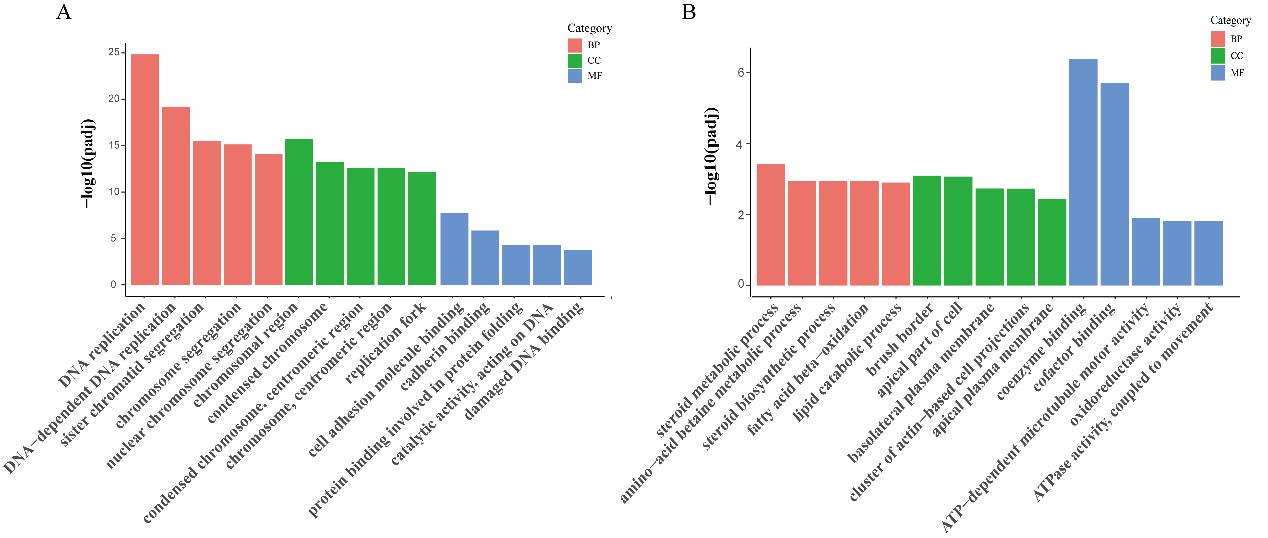
**

The top 5 (A) down and (B) up regulated BP, CC and MF of the differentially expressed genes in 769-P transfected with *shANXA8* compared with *shCtrl*.

BP, biological process; CC, cellular component; MF, molecular function; RCC, renal cell carcinoma.

**Supplementary Figure 4. Full length blots and markers of protein expression of ANXA8 and β-actin in HPTEC, 769-P and 786-O.**

**
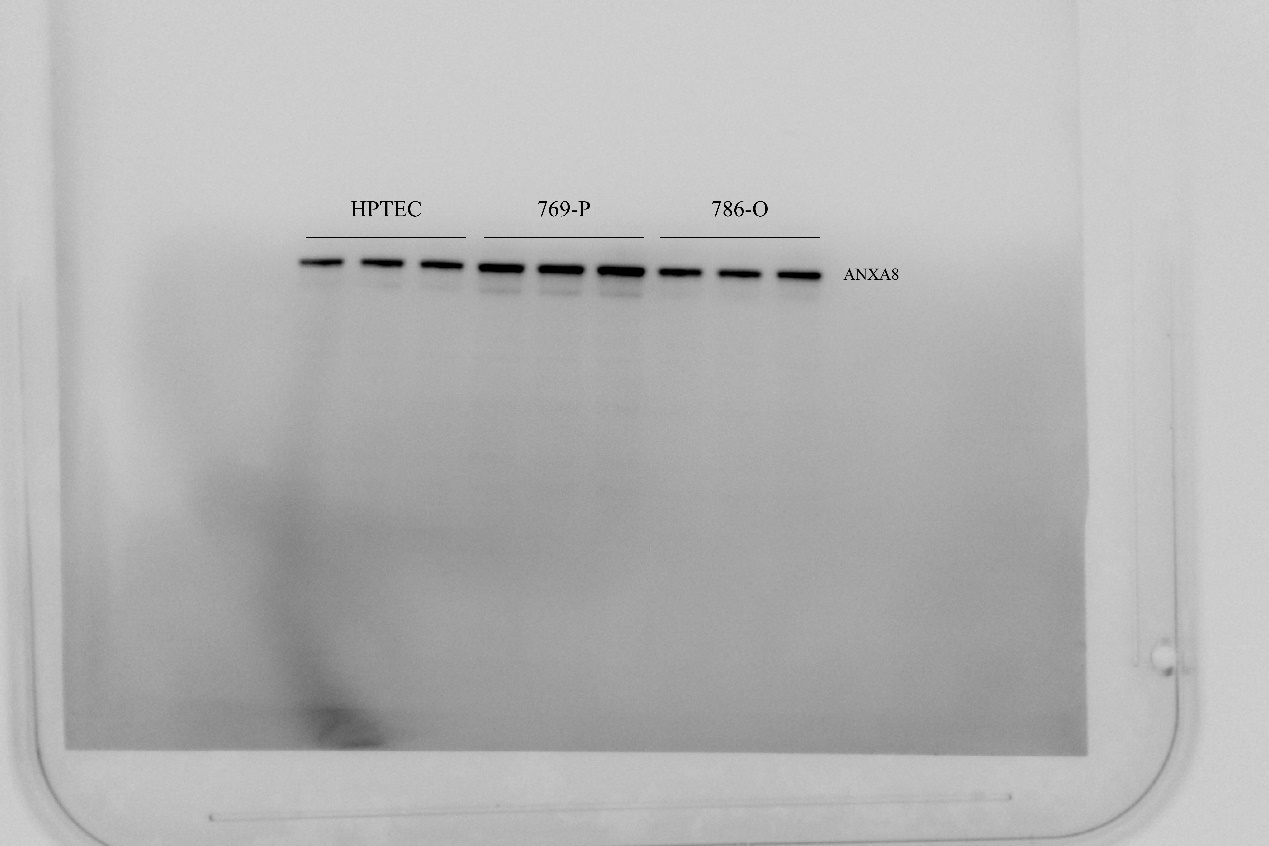
**

**
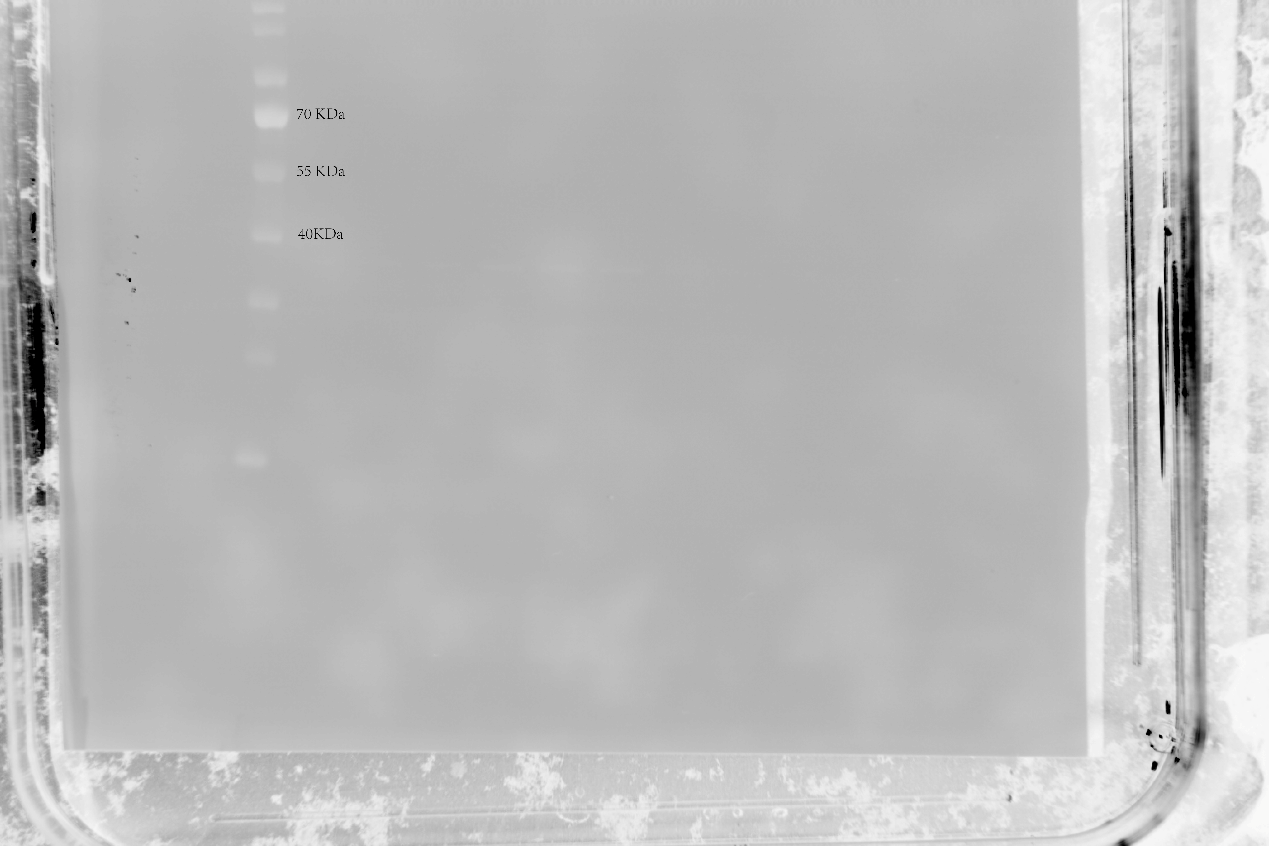

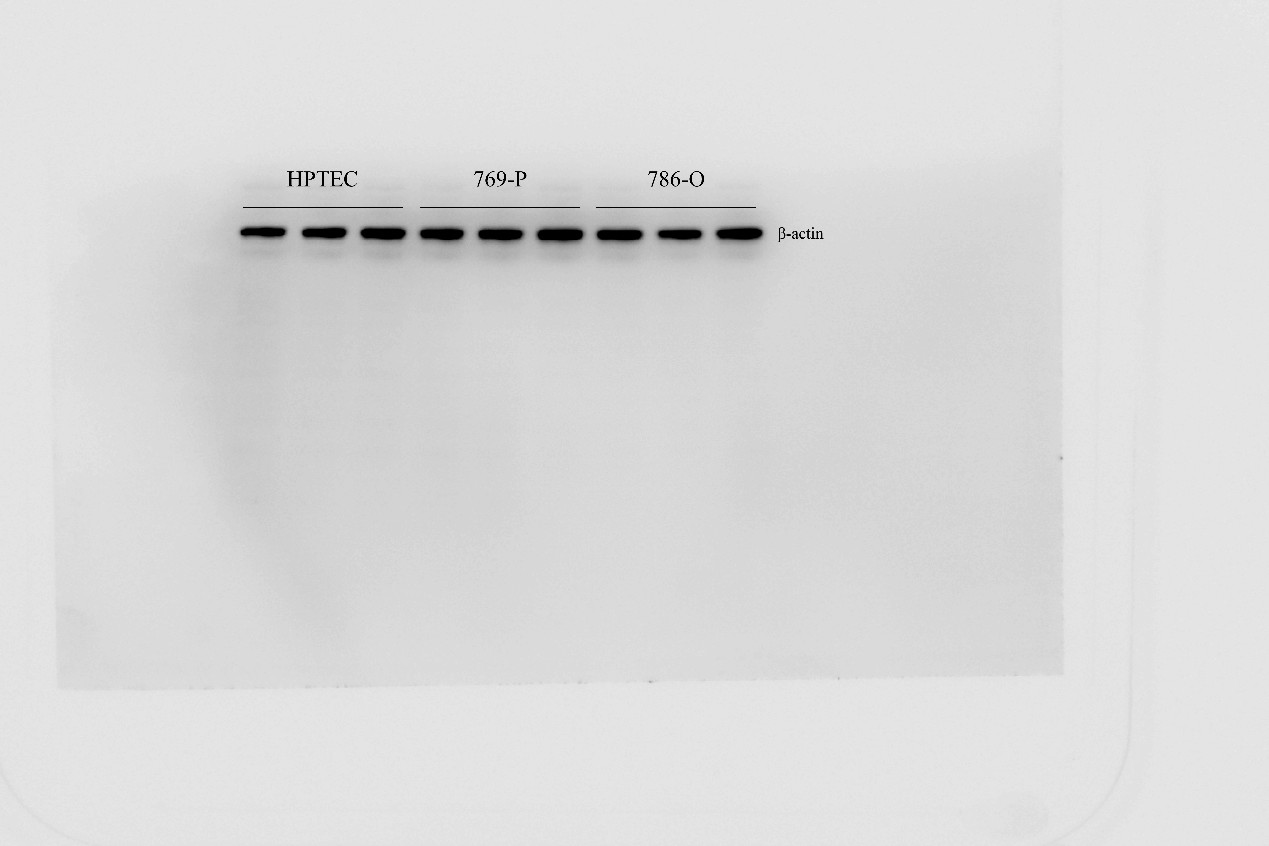
**

**
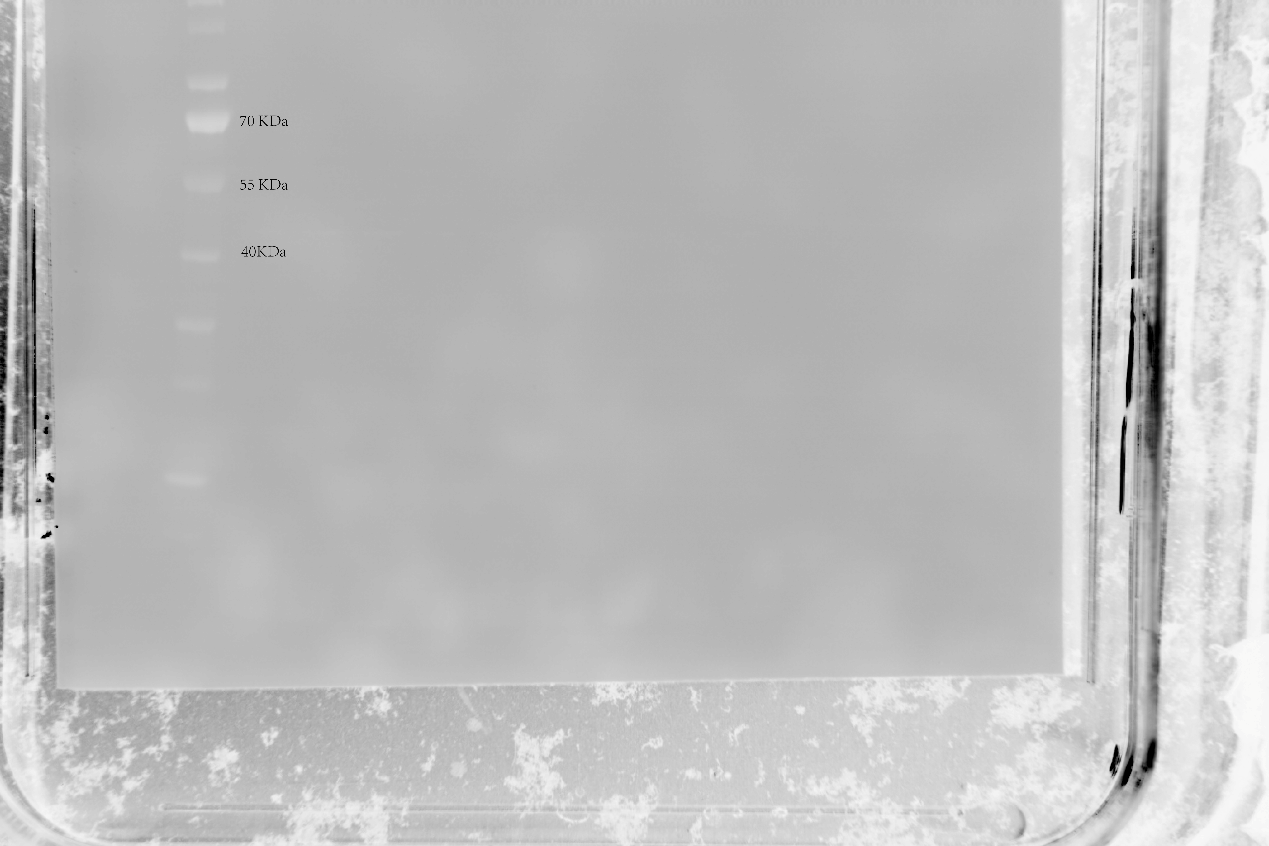
**

**Supplementary Figure 5. Full length blots and markers of protein expression of ANXA8 and β-actin in 769-P transfected with *shCtrl* and *shANXA8*.**

**
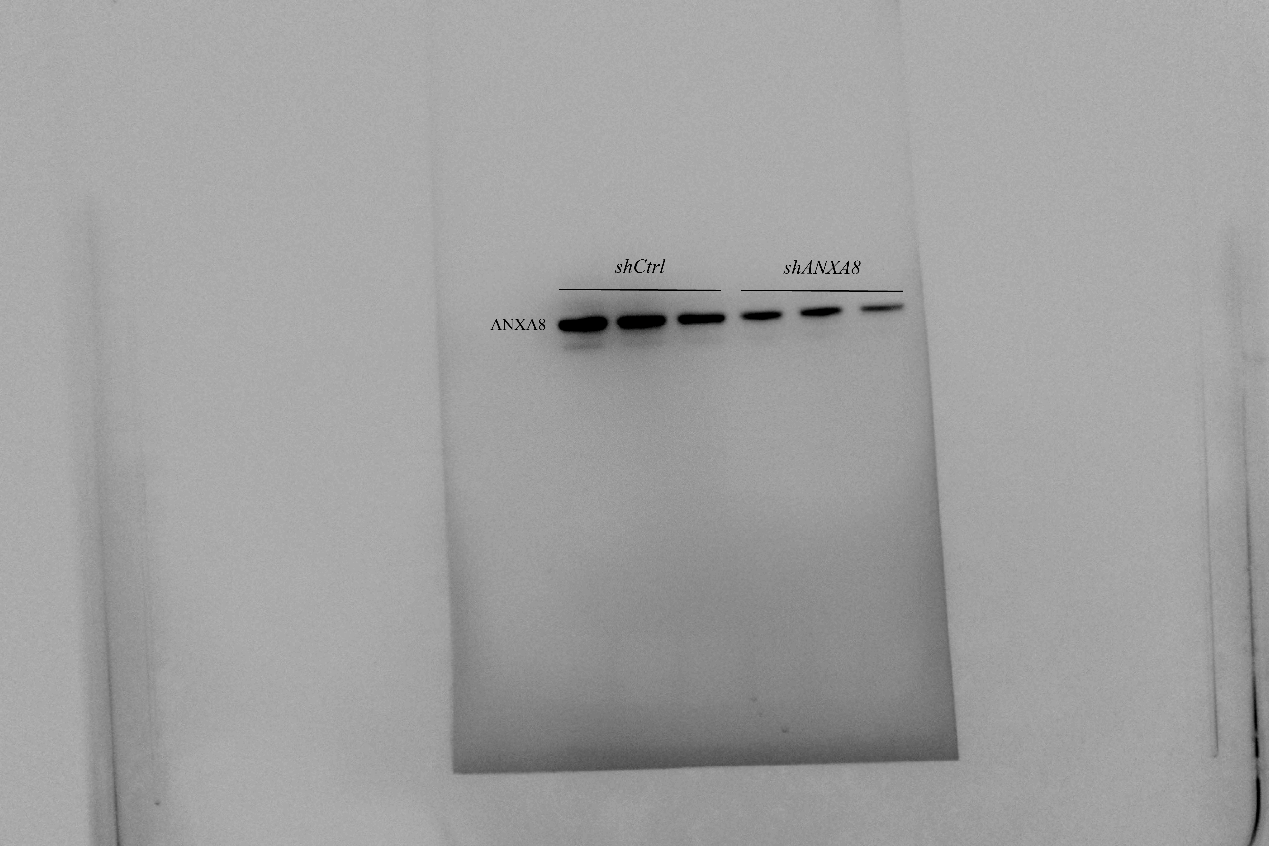
**

**
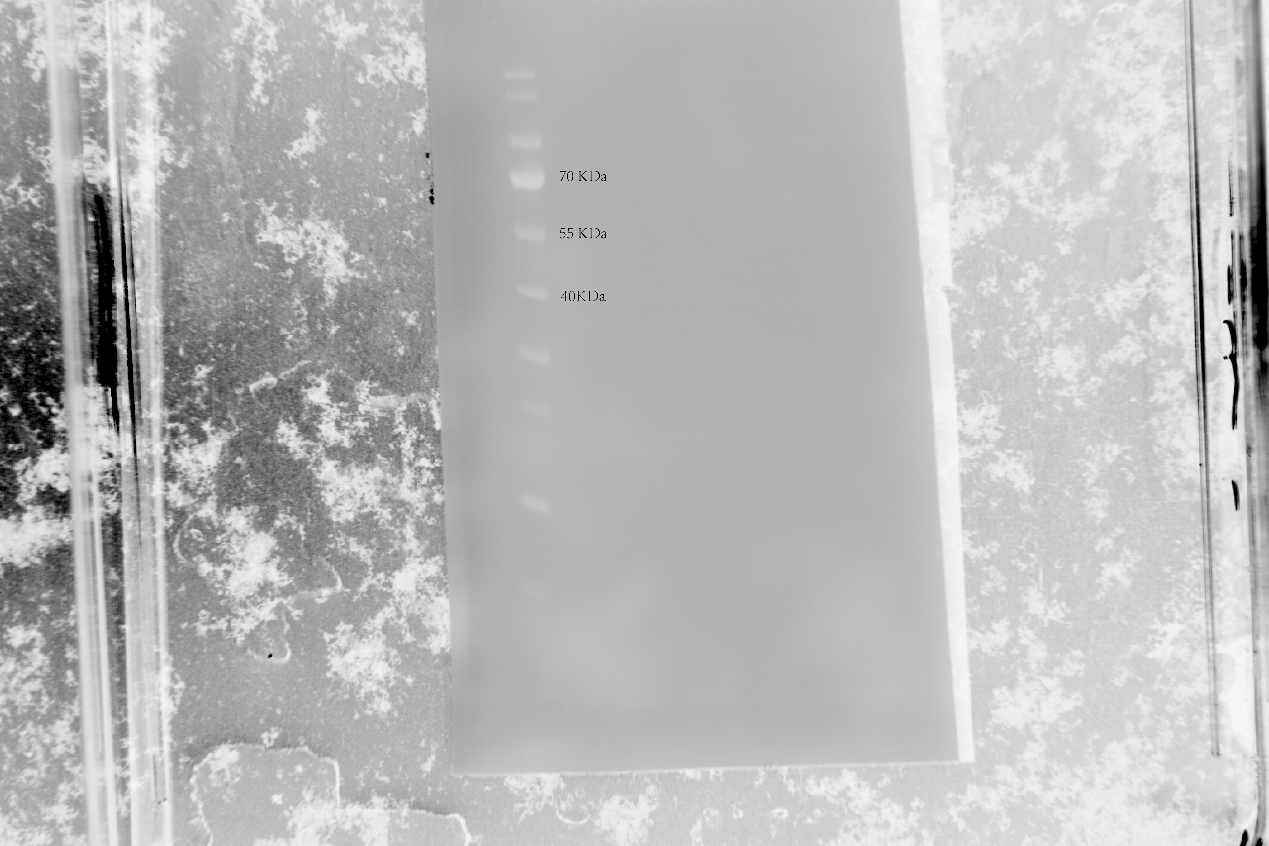
**

**
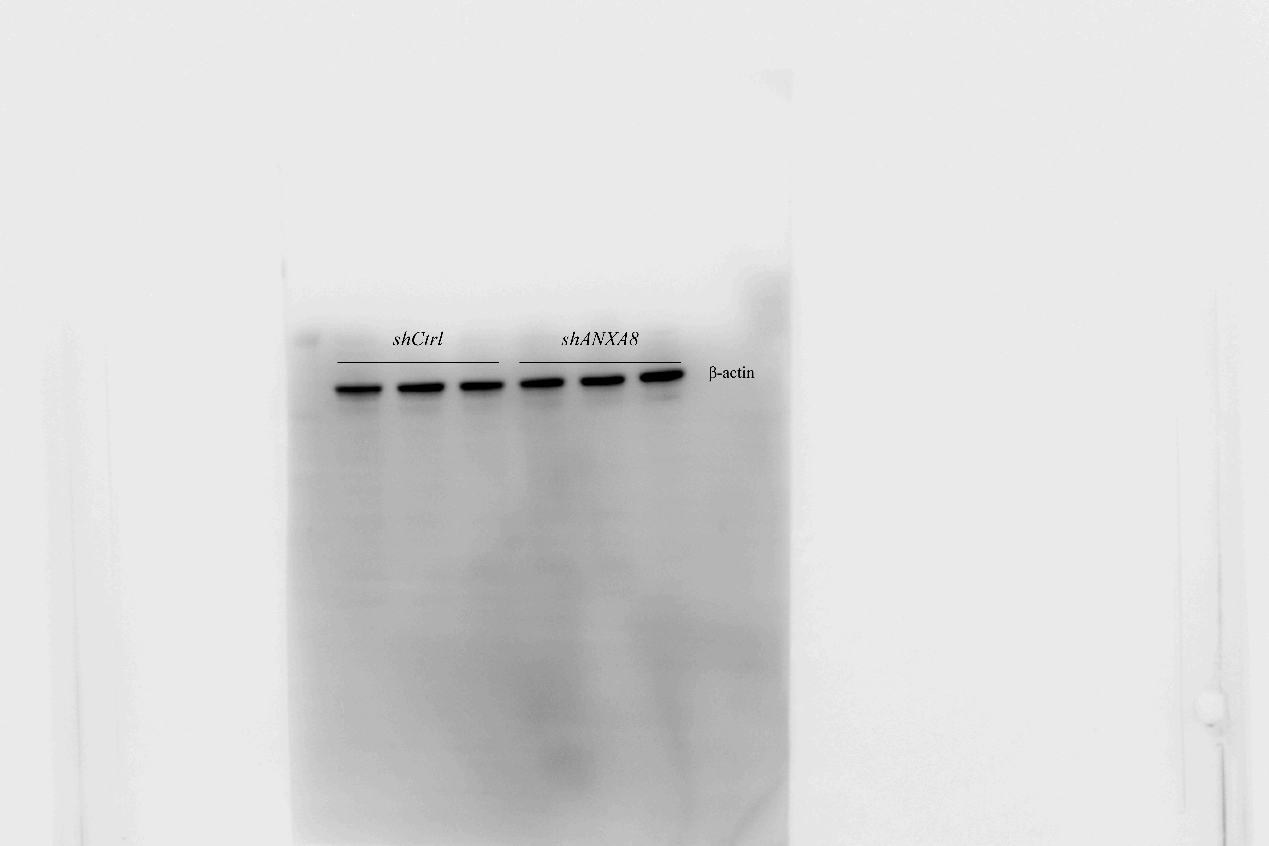
**

**
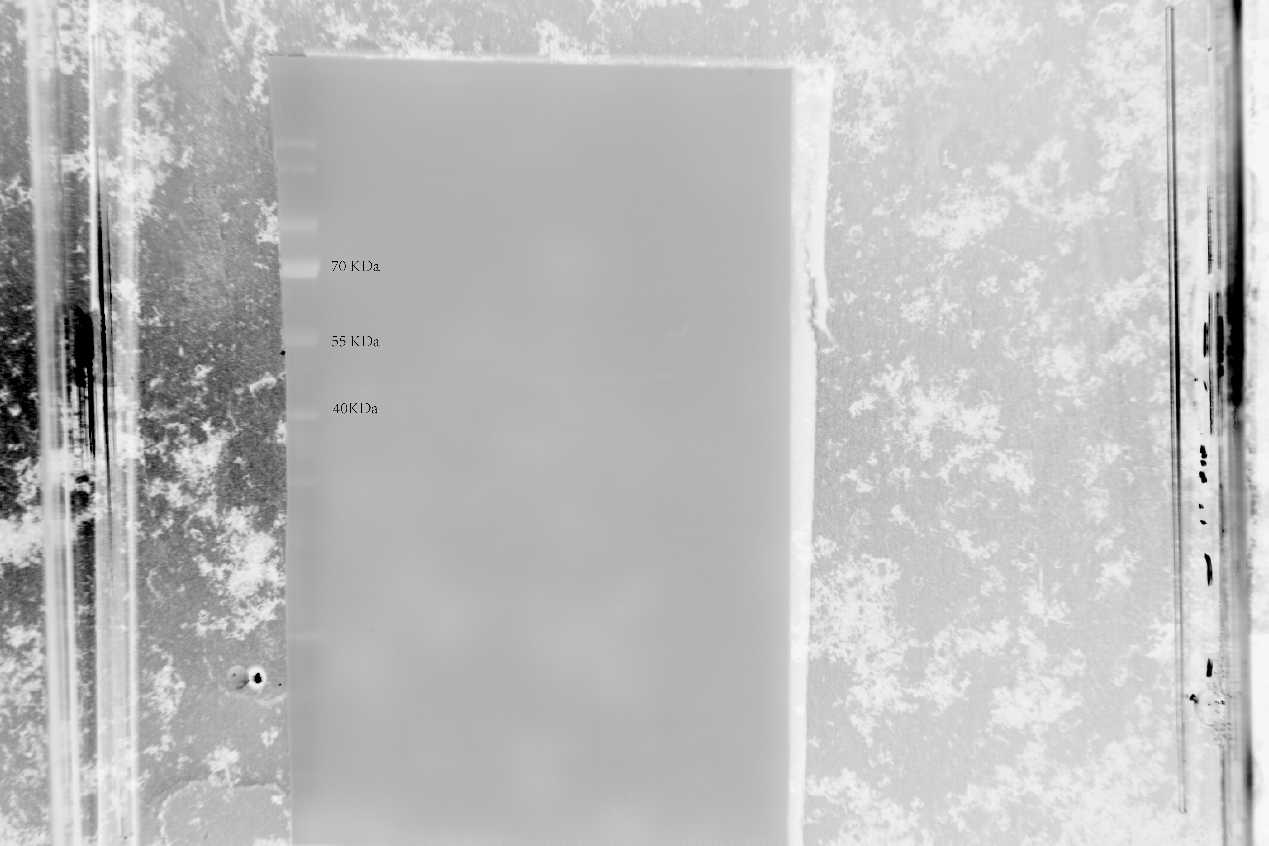
**
